# An experience with the use of WISN tool to calculate staffing in a palliative care hospital in Brazil

**DOI:** 10.1186/s12960-021-00680-2

**Published:** 2022-01-28

**Authors:** Alessandra Pereira da Silva, Mario Roberto Dal Poz

**Affiliations:** 1 National Institute of Cancer José Alencar Gomes da Silva, Rio de Janeiro, Brazil; 2 Social Medicine Institute, University of State of Rio de Janeiro (IMS/UERJ, Rio de Janeiro, Brazil

**Keywords:** Planning, Management, Staffing, Palliative care, WISN

## Abstract

**Background:**

The article describes a healthcare staffing exercise that took place in a Cancer Hospital IV, Brazil’s first public palliative care unit. There are numerous gaps in the literature on specialized cancer staffing. Palliative care is a therapy modality that should begin with the diagnosis of a chronic disease, at which point the personnel must be technically and numerically adequate, as well as well-distributed, to provide coverage of the population that requires this type of care.

**Methods:**

The WISN tool was chosen after a systematic review of the use of workload studies in palliative care, because it fulfills this objective. The WISN method is based on a health worker's workload, was developed in the late 1990s in the health sector and has been field-tested and implemented in several countries. Direct observation was used as the fieldwork approach, which was carried out by 18 research assistants with the assistance of two supervisors. They monitored 60 professionals in seven categories for 2 weeks on weekdays in the morning and afternoon periods: nursing, pharmacy, physical therapy, medical, nutrition, psychology, and social services.

**Results:**

Except for the medical staff, which at the time included additional physicians on loan from a partner institution to address a shortage in this professional group, all categories exhibited overload with WISN ratios ranging from 0.53 to 0.97. The analysis of time spent on individual activities indicated flaws with the services' informal organizations. The authors also noticed a strong emphasis on support activities and a lack of a clear schedule for training and research. The study's findings included a definition of standard activities for each professional group, an analysis and comparison of activities by categories, departments, and work shifts, a standard workload for training and research, and recommendations to include human resources planning as a fundamental part of a national policy for palliative care.

**Conclusions:**

The WISN tool can be used to plan human resources in cancer centers that provide palliative care, and it provides for a variety of analyses that can be combined with other approaches in the literature.

## Background

The literature offers extensive knowledge on important issues in the field of palliative care, such as adequate structure of services, the healthcare staff’s role, multidisciplinary work, inclusion of family members in the treatment plan and management of emotional issues, staff stress and illness, and training and retention of specialists [1–4].

Despite the contribution by the above-mentioned themes to improvement of the supply of palliative care, there is a gap in the literature on the estimation and numerical adequacy of staffing to serve the population [5].

The principal objective of health policy planners is to guarantee available human resources for the population’s access to health services to maintain or improve health conditions. The challenge is to guarantee sufficient human resources for health systems to offer these services [6]. Planning that neglects to estimate the number of staff needed will present a level of incompleteness and could have a negative effect on health outcomes.

Human resources’ central importance exposes the growing recognition of the need for attention to activities in recruitment, training, retention, allocation, and management of these professionals. Evidence also shows that investment in healthcare staff impacts areas, such as education, allowing employment opportunities, facilitating decent work in the formal sector, and fueling economic growth [7, 8].

This finding on the effect and need for sufficient staffing of health systems, however, ran contrary to the availability of healthcare workers to the global health market. According to estimates by the World Health Organization, there will be an increase in the shortage, from 7.2 million workers in the year 2012 to 12.9 million by 2035. Low and middle-income countries such as Brazil are the most affected by epidemics and worker shortage [7].

The aim of this paper is to demonstrate the use of the WISN tool in a reference hospital and to collaborate to establish a palliative care strategy, in Brazil, with an emphasis on human resource workload analysis.

### Context

Considering the relevance of the supply of palliative care in chronic diseases, it is important to note that Brazil has a Strategic Action Plan for Tackling Chronic Noncommunicable Diseases (NCDs) for the period 2011–2022. The Plan’s main objectives are to strengthen the response capacity by the Unified Health System (SUS) and to expand actions in comprehensive care for the prevention and control of NCDs, considered a major health problem, since they account for 72% of the causes of deaths, featuring cardiovascular diseases (31.3%), cancer (16.3%), diabetes (5.2%), chronic respiratory diseases (5.8%) and other NCDs (13.4%). Chronic noncommunicable diseases affect individuals of all socioeconomic strata and have a greater effect on individuals belonging to disadvantaged groups such as the elderly and those with low education and income [9]_._

WHO estimates global cancer incidence in 2030 at 27 million cases, with 17 million deaths, in addition to 75 million persons per year living with the disease [10].

According to international data, in 2012 there were 14.1 million new cases of cancer and 8.2 million deaths. In developing countries, of this estimated total, the incidence was 60% and the mortality reached 70%. Regional, socioeconomic, and cultural inequalities impact the control of risk factors and incidence and prevalence of chronic and degenerative diseases. Such inequalities also interfere in the possibility of individuals receiving timely and adequate treatment, which hinders dealing with avoidable or preventable deaths [10].

In Brazil, the estimate for each year in 2020–2022 points to 625 thousand new cancer cases [11]. The projections for cancer incidence in the global and Brazilian population point to an increase in the need for supply of palliative care in an increasingly older population. In Brazil, there is an increase not only in the absolute number of elderly people but also in the proportion of this group in relation to the Brazilian population. The increase in the elderly population will be even more significant by 2060, when approximately 1/3 of the Brazilian population will be elderly [12]. The discussions on global access to palliative care feature a research agenda moving forward with researchers devoted to projecting the need for palliative care by 2060, based on the concept and methodology of the Commission on Global Access to Palliative Care and Pain Relief. It is thus timely to highlight the importance of adopting a continuing-care approach for the population’s health coverage, including prevention and treatment as part of global access to health, aimed at improving health systems’ performance [13].

The most widely used methods for calculating human resources consider the demand, the population’s needs, government targets for services’ productivity, and indicators of health worker density per a given population. All these approaches to planning human resources for health suffer methodological limitations, that is, there is no specific methodology that solves all of a health system’s problems with human resources [6]. It is thus important to test methods in institutions, services, or departments to share experiences and contribute to improving human resources planning in health.

Planning is widely viewed as a technique disconnected from national health policy. The data that are used are not trustworthy or complete, which hinders adequate information for decision-making. In addition, the focus is on quantitative data, with little qualitative information. Another limitation is that the planning is designed for the medical and nursing professions, ruling out other healthcare workers that participate in the productivity and quality of services provision, based on redesigning the executed tasks. These methods also fail to consider the budget resources earmarked for healthcare and the country’s economic situation. Another important point is that the assumptions on staffing needs should be assessed for accuracy and relevance [14]_._

Staffing for palliative care is an important part of planning in oncology. This is based on cancer’s chronicity, the complexity of available cancer treatments, and global estimates on shortages of healthcare workers [15, 16]. There is thus a need to plan the services supply considering the epidemiological transition, population aging, and urgency of a numerically and technically adequate workforce for this scenario.

An important guideline for staffing in this area of care is the analysis of workload, which impacts healthcare workers’ physical, psychological, and social well-being to various degrees. Progress in academic discussions on healthcare workers’ distress has led to the understanding that the matter is management’s responsibility and poses a challenge for services administration, since poorly staffed healthcare teams, distributed inadequately and with heavy workloads, tend to present higher degrees of suffering, illness, and absenteeism, overloading the workers and affecting the provision of care and the health outcomes [17]. The *Workload Indicators of Staffing Needs* (WISN) tool is thus an option for assisting human resources management and planning. The method orients management changes, contributes to decision-making, identifies work overload, allows reviewing and adjusting the distribution of tasks across the staff, favors improvement of quality of healthcare services, and produces indicators that serve as the basis for establishing future hiring plans [18].

The Cancer Hospital IV, part of the Ministry of Health's National Cancer Institute has a history of important results and is well-equipped, with an outpatient department, urgent care department, physical therapy gym, 53 inpatient beds, and a homecare team. In Brazil there are limitations for guaranteeing access to palliative care when compared to the population’s demand. First, palliative care should be initiated as soon as possible after diagnosis, and the hospital receives patients who no longer have the possibility of cure. Second, the healthcare network should be organized to allow supplying this treatment modality for persons with chronic illness, including cancer, throughout the country and at different levels. Third, estimates point to an increase in the demand for palliative care, which will require various services with healthcare workers trained in this modality.

At Cancer Hospital IV, which is a national reference in palliative care, positive outcomes and structure of varying facilities accredit the hospital for conducting an experiment with staffing estimates that can be used to establish an effective Brazilian palliative care strategy and can also be applied and adapted to different levels of the health system.

## Method

The research comprised a descriptive and exploratory quantitative and qualitative study using the Workload Indicators of Staffing Needs (WISN) tool [18].

For the research, seven professional groups were selected with degrees in nursing, pharmacy, physiotherapy, medicine, nutrition, psychology, and social work. The number of days that a professional does not work in a year was estimated on the basis of hospital standards and absenteeism records, with absences for different reasons, such as vacations, holidays, illness, training, and personal reasons.

Work activities carried out on a regular basis were specified on the basis of literature, hospital records and professional reports, categorized as health service activities carried out by all members of the professional category and periodically reported in statistics; support activities carried out by all professionals of that category; additional activities, which are performed by some members of the category only, and the last two are not recorded in the statistics of the service.

The next step was to calculate the pattern of activity, that is, the time it takes to perform the activity to reasonable professional standards for a trained and well-motivated person in a category. By the time and motion process and observational work sampling, validation of the activity pattern was carried out. For this phase of the study, eighteen research assistants and 2 supervisors were chosen. The prerequisite for becoming a research assistant was to be a health care undergraduate student; social work, psychology, nutrition, and pharmacy students were chosen. Professionals with expertise in coordinating field research in the health area were the supervisors.

Training on the scope of work was provided for the research assistants; ethical and behavioral issues in the hospital setting; the purpose and aims of research; and the limitations of acting directly in activities or processes requiring privacy.

During the period July 22 to August 2, 2019, on weekdays from 7 am to 7 pm, data collection was performed using an electronic excel form, accessible on a tablet for each researcher. The electronic form included filters so that locations and professional groups could be identified with the activities. A 10 min period was considered for the observations. Direct observation covered 60 healthcare workers from different professions and aimed to include the largest possible number of workers while respecting the time limits and required academic quality standards. The inclusion criteria was 5-year experience at HCIV hospital. We thus prioritized participants belonging to health professions working in outpatient services, inpatient care, homecare, emergency, the physical therapy gym, and the pharmacy.

In 2018, the productivity data for the hospital were: 2034 hospital patients; 1332 deaths; 53,557 nursing procedures; 15,666 physical therapy procedures; 2903 24-h observation emergency care. Physicians' outpatient consultations (7629), nurses (28,615), physical therapists (1689), nutritionists (14,796), psychologists (4891), social workers (6512), nurses' emergency unit consultations (2840), nutritionists (1266) and physicians' consultations (2904). Home visits carried out by nurses (6366), physicians (1649), social workers (1585), psychologists (693) and physical therapists (237), were taken from the hospital's information system.

The calculations defined by the formulas provided in the WISN manual were carried out with the aid of software, a specific technological resource for the use of the WISN process, created and made available by the WHO, to quantify the workload and to evaluate the adequacy of the hospital dimensioning. Based on the last career salary table for each category, the annual salary was calculated. The tool included data representing the average number of absences for each occupational group for all types of leave.

The next step was to fill in the statistical data available on the activities carried out within the facilities. Information on productivity from the information and planning system of INCA, maintained and updated by each hospital, was used.

The activities selected were: home visits; outpatient consultation; death services; hospitalization procedures; emergency room consultation; inpatient services; nursing and physiotherapy procedures.

Activities with statistical findings not available were chosen according to the highest relative frequency of occurrence and varied between categories. Activities such as writing medical records, preparing reports, requests for supplies and medicines, prescriptions, drug scheduling, minutes of meetings, follow-ups and those of a similar nature were included in the content registered in hard copy papers and the electronic system. Research and training activities were included in this group.

The method stipulated that 15 percent of the workload, or 6 h/month, would be the standard for all categories to conduct research work, according to what was reported in the fieldwork and because there is no normative guidance to specify a percentage of the workload for this activity. The individual activities were related to management activities—preparation of schedules, meetings with the Directorate, administrative activities and visits to the hospital beds.

The calculation of the standard time in minutes for the execution of each activity was extracted from the database formatted from the excel form used in the fieldwork.

The next step was the calculation of workload standards, which show the amount of work for each activity that a professional can perform in a year. This information is automatically calculated by the WHO software with calculations about the average number of days worked per year; the apportionment of the time of the activities in different measurement units; the percentages of workload and the ratio between the number of existing, surplus, and needed professionals.

The analysis and interpretation of the results were performed by comparing data on categories, place of work, work schedules, training, research, displacements, administrative and personal activities.

## Results

The integrative review revealed the lack of palliative care staffing literature.

A list of coded activities for social workers, nurses, pharmacists, physical therapists, physicians, nutritionists, and psychologists was prepared for the fieldwork. Direct observation was conducted with 60 professionals in various sectors, and some were observed more than once.

In the database, a total of 4292 valid records were identified for analysis. After adjusting the database records, we counted 1019 health service records, 1095 support records and 596 additional activities performed by the seven professional categories in the five services defined. The other records were for meals, displacements, physiological functions, and personal activities, work hours beginning and ending. The activities reported were numerically adequate for a segmented analysis to be feasible, with the possibility of comparing programs, categories and job shifts.

**Fig. 1:**
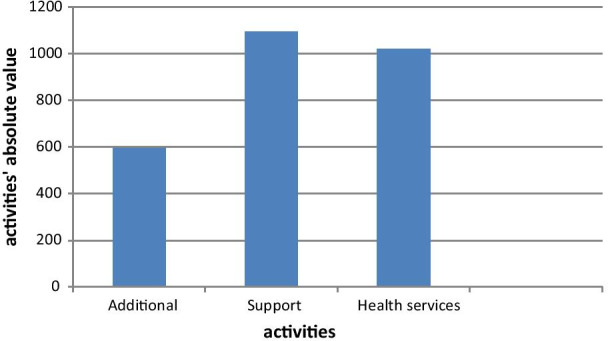
Number of health services, support, and additional activities in the Cancer Hospital IV, INCA, Brazil, 2019

Figure 1 shows a predominance of support activities, which highlights the relevance of the activity’s nature when planning staffing.

Twenty-four nurses were observed in emergency care, outpatient care, home care, and hospitalization services. Nursing support activities (398) exceeded the sum of health services activities (298) and additional activities (89).

Social Work activities were observed in the outpatient clinic, home care, and hospitalization. Ten social workers were followed up. Health services activities (138) and support activities (141) are at similar levels in terms of the absolute number of observations. The additional activities (81) observed involved administrative, training, research, and management activities and represent 22% of the observed activities.

Eighteen physicians were observed in emergency care, outpatient, home care, and inpatient services. Compared to the health services activities (173), the number of support activities (167) is high and demonstrates the demand of the work done by the physician when he/she is not in direct patient care. The additional activities of the category (89) were administrative, training, research and management and represent 20% of the observed activities.

Nutrition activities were recorded in the outpatient and inpatient units with the observation of 5 professionals. Support activity work (80) and additional activities (63) stand out in relation to health services activities (23) Unlike other categories, research and training activities were found to stand out in the additional activities category.

The Psychology activities were observed in the outpatient clinic, home care, and hospitalization, with the follow-up of 6 professionals. This was the only category in which, because of the large number of training tasks, additional activities (152) stood out in comparison to the other activities. Support activities (101) represented the lowest volume in absolute numbers, which indicates that the category reconciles the agenda with health services activities, even with a large administrative burden (150) and a significant workload of training activities.

In home care and pharmacy, the activities of 10 pharmacists were observed. Compared to the other occupations analyzed, the number of additional activities observed (22) showed a low amount of time in training and research. Although support activities (107) stand out, the number of health services activities (95) points to an intense pharmacy clinical activity and a closer interaction between the practitioner and the patients and their families.

The physical therapy activities were observed mostly in the gymnasium, where four physical therapists work. Some observations were made on the inpatient floors, when there was demand. Health services activities (138) represented the majority of activities, while in support (101) and additional activities (104), there was a balance in the distribution of administrative, management, training and research activities.

**Fig. 2:**
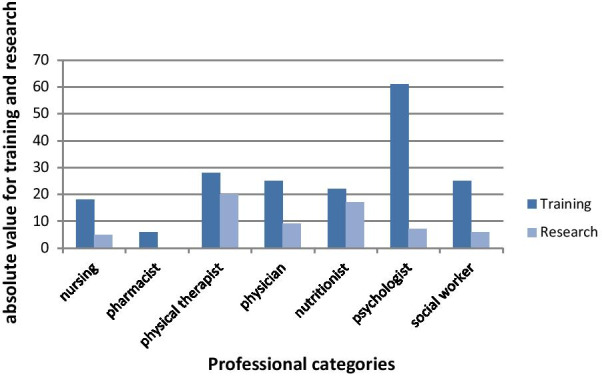
Proportions of training and research activities in the Cancer Hospital IV, INCA, Brazil, 2019

As shown in Fig. 2, the time dedicated to training and research activities does not point to an institutional pattern.

**Fig. 3:**
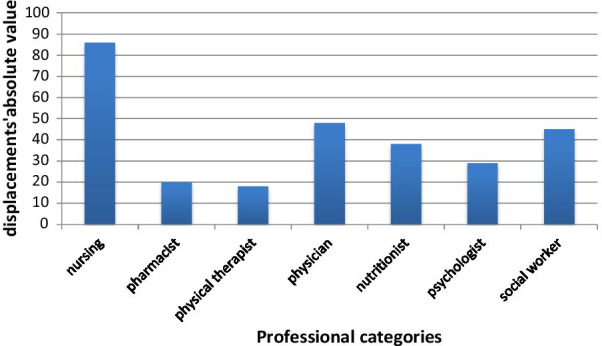
Number of displacements by healthcare workers in the Cancer Hospital IV, INCA, Brazil, 2019

Another focus of the study’s observation is healthcare workers’ displacements. This included internal displacement in the unit, home visits, and visits to other INCA’s hospitals and other institutions. Figure 3 shows the number of displacements for each category.

286 displacements were registered, 90% of which were made on the Cancer Hospital IV premises. The internal displacements have several purposes, such as: to attend patients on the floors; to go to meetings; to search for printed material; to search or return medical records to the file; to visit different patients on the same inpatient floor and to accompany patient transfers. According to Fig. 3, of the total displacements observed 30% were from nurses; 17% from physicians; 15.8% from social workers; 13.3% from nutritionists; 10.2% from psychologists; 7% from pharmacists, and 6.3% from physical therapists. The displacements for home visits were about 9%. The percentage of displacements in relation to health services, support and additional activities performed represented 5% for physiotherapists; 7% for psychologists; 8% for pharmacists; 10% for nurses; 11% for physicians; 12% for social workers and 22% for nutritionists.

The current staffing data were reported by the Cancer Hospital IV, and the comparison was performed with the estimate by the WISN software. According to Table 1, the result showed a shortage of all the professional categories except physicians.

**Table 1:** Comparison of current and recommended staffing

Type of staff	*A* Existing staff	*B* Calculated requirement	*C* Difference in staff (*A*–*B*)	*D* WISN ratio (*A*/*B*)
Social worker	8	10	− 1.81	0.82
Nurse	45	46	− 1.43	0.97
Pharmacist	7	12	− 5.21	0.57
Physical therapist	5	7	− 2.18	0.70
Physician	20	17	2.94	1.17
Nutritionist	8	10	− 1.6	0.83
Psychologist	6	11	− 5.36	0.53

According to the WISN tool, when the ratio in column D of Table 1 is 1, there is no overload, since there is a balance between the current staffing and the service’s need. The lower the ratio, the higher the workload. Thus, the result points to a high work overload for all the categories except physicians, and is highest for psychology and pharmacy.

The categories with the largest shortage were psychology and pharmacy, with a lack of approximately five workers each. Physical therapy showed a shortage of more than two physical therapists, while social services, nutrition, and nursing showed shortages of up to two staff members each.

## Discussion

INCA has a platform called Normatiza, with the norms referring to professionals; however, the information obtained in Normatiza and the reports of professionals from the categories studied, already in the first meetings, were not enough to draw up a reliable list of the activities that are performed on a daily basis. The researchers had difficulty in detailing and explaining several activities, since many are interpreted as embodied or automated, and many are interrelated and performed simultaneously.

The fact that the volume of support activities exceeded that of health services activities underlines that to maintain direct care for patients and families with quality requires a workload for indirect and administrative activities to assist the organization of services and guarantee the continuity of care. For example, for lower complexity care, the healthcare worker reads information in advance that the team has recorded in the patient’s file, consults the prescription, and plans the necessary material for procedures. After the direct work with the patient, the worker records the care provided on the patient’s file, discusses the clinical case with the other team members, records his or her own activity, and updates the treatment plan for next round. The tasks preceding and succeeding the direct care are relevant and represent an important workload.

In addition to the patient care activity, the healthcare staff perform research activities aimed at scientific and technological development of new strategies for cancer diagnosis and treatment. INCA offers training activities that include distance education, medical and multidisciplinary residencies, Master’s, PhD, and postdoctoral programs [19]. Although there are specialized staff members in the respective areas, the healthcare workers that provide patient care also conduct these training and research activities. The adequate proportion of time for the workers that conduct training and research activities lacks a standard definition. In the current study, for the workload analysis, we stipulated a time for these activities, since the results do not point to an institutional standard for this purpose, as shown in Fig. 2’s data.

Dedication to training and research activities accounted only 249 records for these activities, 74% were for training and 26% for research activities, distributed across all the professional categories. There is no standard orientation by either the institution itself or the Federal government concerning the organization, targets, and evaluation of the training and research responsibilities of patient care health staff. Thus, training activity is shaped by the demand from the department of training, and research depends on the personal motivation and organization of the staff members in relation to the time dedicated to research. One of the benefits of this study was thus to shed light on the discussion concerning the need to stipulate necessary time for research and to identify how training activities impact workload, although they are not represented as productivity per se. It became clear that despite the organization of statistical data, there is an important gap in the availability and public sharing of this information.

The displacement results from the hospital’s architectural layout, home visits, and the flow of staffers to perform their responsibilities. A total of 286 displacement were recorded, 90% of which within the Cancer Hospital IV. Internal displacement involves various movements, such as visiting patients on the four floors, attending meetings, collecting printed material, picking up or returning patient files to the hospital archives, visiting patients sequentially on the same floor of admission, and accompanying patient transfers. Home visits accounted for 9% of the displacement in planning.

Displacement as a proportion of health services, support, and additional activities, according to professional categories, accounted for 5% for physical therapy, 7% for psychology, 8% for pharmacy, 10% for nursing, 11% for medicine; 12% for social services, and 22% for nutrition.

This time for the healthcare worker impacts productivity and the duration of other activities and also serves as an indicator when calculating staffing, as a factor that influences workload.

Medical leave due to illness was the main cause of absenteeism (1674 days) and mourning leave and leave to accompany sick family members accounted for 5% of this total. No unjustified absences were recorded in the period.

Absenteeism is a challenge for organizations and for managers, because it is a multi-causal phenomenon, which can have its origins in working conditions; in management and leadership styles; in the possibility of human resources participation in decision making, and in daily professional relationships. These and other causes need to be identified, understood, and worked on by the organization.

The literature associates high rates of absenteeism with Burnout Syndrome, turnover, and decreased productivity [3]. Absenteeism can trigger the collective illness of teams, because the lack of human resources leads to a greater effort by others to provide care, generating overload and physical and psychological consequences for the health of professionals.

The staff at INCA and all its hospitals consists of public employees who joined the institution’s workforce through public competition. The INCA does not have the administrative autonomy to hire personnel and adjust its staffing. Although the current study does not guarantee immediate adjustment of the working conditions, it can assist the administration and staff in improving the processes and arguing (on a solid theoretical basis) for redesigning the staffing. For example, the current medical staff includes members of INCA’s own public employees, plus physicians on loan from the Ministry of Health office located in Rio de Janeiro. The conclusion, based on the parameters in this analysis, is that the institution’s own staff of physicians (public employees) would be numerically insufficient without the additional physicians on loan from the Ministry of Health. Another significant point is that physicians’ absenteeism was the highest among the healthcare professions, which may lead to an overload on some physicians even in an apparently sufficient scenario.

The overload on social services, nutrition, nursing, and physical therapy, where the difference between estimated and current staffing was close to or less than two workers, can be managed with revision and redistribution of activities; identification of overlapping tasks, and the establishment of detailed annual production targets. Importantly, better documentation of activities produces more trustworthy results with this method. Thus, the observations do not rule out the need for a numerical review of the staff, a procedure that makes the productivity data more robust.

Pharmacy was the professional category with the second highest absenteeism rate, suggesting a correlation between work absences and workload. Alongside psychology, pharmacy was one of the two categories with the highest overload. In addition, the pharmacy staff also cover the needs of a hospital unit at the same address, specialized in breast cancer treatment.

Besides the more urgent need for a review of staffing, management measures also need to be adopted to avoid an even greater overload: review of targets, adjustment of the supply of care, considering the demand and installed capacity, and greater flexibility of the work hours according to the work schedule.

### Recommendations and lessons learned

The healthcare workers need to have specific skills and qualities as well as knowledge of what they will face in the area of palliative care. Incomplete and mismatched information on staffing in a health service or system hinders planning health promotion measures to benefit the staff themselves. In other words, knowing who these workers are, their training, their geographic area of work, and their skills provides backing that favors caring for those who care for the patients on a daily basis, as well as helping ensure adequate staffing in relation to workload.

The WISN tool works with the available data in the statistics of health institutions or services. Thus, without centralization and monitoring of the information to guarantee trustworthiness, the method’s result will be less precise, which does not rule out its application.

Besides the productivity data, knowledge of the absenteeism rates per category, specifying the cause of absences, helps not only to feed the tool’s data but also allows a diagnosis for the administration to implement health promotion measures for the staff.

Although the method indicates stipulating four health services activities, three support activities, and three individual activities per category, if the number and variety of statistical data are improved every year, the analysis will be more robust, to the extent that it reveals peculiarities in staff productivity.

The more the number of professional categories analyzed, the easier it is to understand how the services’ staff functions. For a more in-depth analysis, staffing estimates can be complemented with other methods [6, 14, 15].

All the internal and external activities should be covered in the observation. External activities such as home visits impact the workload and the healthcare workers’ work dynamics. A relevant point for the support activities is that both in the literature and in the current study, they outweighed the health services activities. Thus, planning based on activities such as consultations, procedures, and various treatments and that fails to consider the necessary time for support activities will suffer a conceptual error. Recognition of these activities as part of the list of staff responsibilities allows staffing predictions and distribution that are closer to the reality and that lend visibility to the issue of backstage work that impacts the quality of care.

Training of research assistants is crucial for the fieldwork to occur under a single guideline, with occasional problems solved in timely fashion. The study revealed the advantages of using well-trained students with direct supervision by experienced professionals. Veteran professionals may make their own interpretations of what is seen, which escapes the purposes of direct observation, introducing biases into the observations or mistakenly transmitting to the observed workers the idea of a qualitative assessment of the care provided. With students, the training has to be more rigorous, but the receptiveness tends to be more favorable.

## Conclusion

Health services organization and planning involve measures aimed at positively and directly impacting the population’s health coverage and the supply of quality services. Although Brazil’s National Cancer Policy has been improved [20], palliative care lacks its own policy for this modality to be included in the network to guarantee access and coverage at all levels of care.

The tool allowed observing details in the work process that are not perceived as a source of work overload. For example, lunch breaks are usually interrupted or shortened by some work activity, overlapping of tasks is also routine, which even hinders some observational records, annotations on the patient chart after home visits are performed in the car, and the use of personal cellphones to contact other team members is an important communication tool. These observations do not represent mistakes or failures by the teams, but the context in which they occur need to be identified, acknowledged, and examined when assessing staff workload and setting targets for the service. The impact on the calculation resulting from the projection for personal activities such as lunch breaks, rest, and physiological needs is relevant for staffing.

An annual review of the service’s structure is recommended to assist the administration’s decisions on internal staff redistributions, flexibilization of schedules insofar as possible to minimize workload, implementing support programs to reduce workload, and backing arguments for adequate staffing composition.

With the results obtained in the field research, we conclude that the WISN tool is applicable to cancer institutions providing palliative care and produces information that allow more in-depth future studies according to services or professional categories, including in association with other established methods such as those used by nursing. The field analysis allowed drafting a complete document with all the activities for each professional category, including the relative frequency of the activities and a comparison with the activities described initially in the literature and by the healthcare workers, demonstrating the importance of organization of activities in a health service. Caring for those who provide care assumes planning, tackling obstacles, and decision-making and demands based on data. The WISN tool proved pertinent for analyzing workload, calculating staffing, assisting the administration, and backing a policy for palliative care based on human resources planning and health services organization.

In the case of the hospital analyzed, the study provides elements that contribute to the development of a palliative care strategy that recognizes the centrality of human resources in the health care system and the impact of workload on the provision of health care to the population, in addition to the applicability of the WISN tool in a palliative care team.

## Data Availability

The datasets used and/or analysed during the current study are available from the corresponding author on reasonable request.
